# Emotional expression recognition and attribution bias among sexual and violent offenders: a signal detection analysis

**DOI:** 10.3389/fpsyg.2015.00595

**Published:** 2015-05-07

**Authors:** Steven M. Gillespie, Pia Rotshtein, Rose-Marie Satherley, Anthony R. Beech, Ian J. Mitchell

**Keywords:** sexual offender, antisocial, facial expression, emotion, signal detection theory (SDT)

## Abstract

Research with violent offenders has consistently shown impaired recognition of other’s facial expressions of emotion. However, the extent to which similar problems can be observed among sexual offenders remains unknown. Using a computerized task, we presented sexual and violent offenders, and non-offenders, with male and female expressions of anger, disgust, fear, happiness, sadness, and surprise, morphed with neutral expressions at varying levels of intensity (10, 55, and 90% expressive). Based on signal detection theory, we used hit rates and false alarms to calculate the sensitivity index *d*-prime (*d′*) and criterion (*c*) for each emotional expression. Overall, sexual offenders showed reduced sensitivity to emotional expressions across intensity, sex, and type of expression, compared with non-offenders, while both sexual and violent offenders showed particular reduced sensitivity to fearful expressions. We also observed specific effects for high (90%) intensity female faces, with sexual offenders showing reduced sensitivity to anger compared with non-offenders and violent offenders, and reduced sensitivity to disgust compared with non-offenders. Furthermore, both sexual and violent offenders showed impaired sensitivity to high intensity female fearful expressions compared with non-offenders. Violent offenders also showed a higher criterion for classifying moderate and high intensity male expressions as fearful, indicative of a more conservative response style, compared with angry, happy, or sad. These results suggest that both types of offender show problems in emotion recognition, and may have implications for understanding the inhibition of violent and sexually violent behaviors.

## Introduction

Facial expressions of emotion serve a critical role in human social and emotional behavior. Earlier studies (Ekman and Friesen, 1971; Ekman, 1972, 1992a,b, 1993), as well as more recent ones (Matsumoto, 2001; Elfenbein and Ambady, 2002; Ekman and Friesen, 2003), provide evidence for six basic emotions that are universally recognized across cultures. It is argued that these expressions, referring to anger, disgust, fear, happiness, sadness, and surprise, are central to social interaction in three ways: they provide information about the emotions and intentions of the expresser, they evoke responses in the perceiver, and they provide incentives for desired social behavior (Keltner, 2003).

Perhaps unsurprisingly, problems in recognizing others facial expressions of emotion can lead to a breakdown in social and emotional responding, with difficulties observed in relation to autism (Gross, 2004), attention-deficit hyperactivity disorder (Singh et al., 1998), schizophrenia (Trémeau, 2006), anxiety disorder (Easter et al., 2005; Palm et al., 2011), and psychopathy (Dawel et al., 2012). Difficulties in social and emotional responding also extend to individuals with antisocial behavior who show a failure to adhere to social rules. However, offending behavior can manifest in different ways, with one of the most common distinctions made between those who commit violent offenses, and those who commit sexual offenses. The aim of the current paper therefore was to investigate differences in the emotion recognition abilities of sexual and violent offenders, and non-offending controls.

In support of problematic social-cognitive functioning in antisocial populations, a meta-analysis by Marsh and Blair (2008) found a specific impairment in fearful expression recognition among instrumentally violent populations. Similarly, Hoaken et al. (2007) observed deficits in facial expression recognition among violent offenders, including those convicted of sexual offenses, compared with non-violent offenders and non-offender controls. However, impairments in emotion recognition may not be specific to violent offenders. For example, Robinson et al. (2012) found poor recognition of anger, fear, sadness, and disgust in a sample of 127 Scottish male prisoners, while Bagcioglu et al. (2014) found impaired disgust recognition among individuals with a diagnosed antisocial personality disorder. These difficulties have also been shown to extend to problems recognizing dynamic bodily expressions of emotion, with impaired recognition of fearful bodily expressions observed among violent offenders relative to non-offender controls (Kret and de Gelder, 2013). Furthermore, impaired facial emotion recognition has also been noted in relation to psychopathic traits, characterized by a lack of remorse or guilt, and a deceitful and manipulative interpersonal style (Hare, 2003), among adult offenders and non-offenders (Blair et al., 2004; Montagne et al., 2005; Dolan and Fullam, 2006; Dawel et al., 2012), and among children with psychopathic tendencies (Blair et al., 2001; Dadds et al., 2006; Fairchild et al., 2009; Jones et al., 2009; Viding et al., 2012).

As well as reduced accuracy, specific patterns of emotion attribution biases have also been noted in relation to antisocial behavior. For example, Dadds et al. (2006) examined errors during an emotion recognition task in a sample of school children with varying levels of antisocial behavior and psychopathic traits. Results showed that antisocial behavior was associated with poorer performance for recognizing neutral faces, which were most often mistaken as angry. These findings are consistent with a hostile attribution bias among antisocial and aggressive children, whereby malevolent intent is assigned to neutral expressions (Dodge and Pettit, 2003). Furthermore, Dadds et al. (2006) showed that psychopathic traits in children were linked with classification biases for fearful expressions, with fearful faces most often confused with neutral or disgust expressions. Similar errors have also been highlighted by Leist and Dadds (2009) in a sample of antisocial youth, where the most commonly made errors for neutral expressions were sad or angry, which accounted for 44 and 40% of errors, respectively.

Despite various links between antisociality and facial expression recognition, there is a paucity of research that investigates emotion recognition in relation to sexual, compared with violent offending. As noted by Leist and Dadds (2009), the relative impact of different etiological factors can vary for different subtypes of antisocial behavior (Wootton et al., 1997; Dadds and Salmon, 2003; Viding et al., 2005). Thus, different etiological factors may be related to sexual and violent offending. Hanson and colleagues have shown that sexual offense recidivism can be most accurately predicted on the basis of deviant sexual preferences, sexual preoccupations, and general self-regulatory problems (Hanson and Busière, 1998; Hanson and Morton-Bourgon, 2005). However, these factors are of relatively less importance for predicting violent recidivism. Different approaches to sexual and violent offending are also reflected in the use of different treatment programs for these two subtypes of offender. For example, specific treatment targets outlined for sexual offenders include socio-affective functioning, self-management, distorted attitudes, and sexual interests (Hanson and Harris, 2000, 2001; Thornton, 2002; Webster et al., 2006). However, despite differences in etiology and treatment approaches, the extent to which these two subtypes of antisocial behavior are characterized by divergent patterns of social-cognitive impairment remains unclear.

The few studies that have addressed facial expression recognition in sexual offenders have identified problems in distinguishing between fear and surprise (Hudson et al., 1993), as well as impaired processing of negative expressions, including anger, disgust, and fear (Gery et al., 2009). However, these results are tempered by failures to measure relevant personality traits, or by designs that fail to contrast the performance of sexual offenders with that of non-sexual, violent offenders, as well as healthy controls. For example, Gery et al. (2009) compared the performance of sexual offenders with offenders imprisoned for theft or fraud. These offenses, however, do not involve aggressive or forceful contact with a victim, and may therefore be characterized by different patterns of social cognitive impairment. As such, the ways in which the performance of sexual offenders compares with that of other non-sexually violent offenders remains unknown.

In this study we aimed to examine recognition accuracy for the six basic emotional expressions (anger, disgust, fear, happy, sad, and surprise), among sexual and violent offenders compared with healthy controls. Stimuli included images of human facial expressions that were varied in terms of both the intensity of the expression, and the sex of the model displaying the expression. Varying the intensity of expressions allowed us to examine emotion recognition for more life-like representations of each expression (Adolphs and Tranel, 2004), and made the task more sensitive to subtle differences in the processing of emotional expressions (Calder et al., 1996). Varying the sex of the model allowed us to examine recognition impairments that may be specific to male or female expressions, and allowed us to account for differences in the expressivity of male and female faces (Schwartz et al., 1980; Brody and Hall, 1993), and the ease with which male and female expressions can be recognized (Hess et al., 1997). The importance of examining responses for male and female stimuli is highlighted by Kret and de Gelder (2013) and Kret et al. (2011), who found evidence for differential processing of threatening male and female emotional stimuli. Given that the processing of facial affect may also be influenced by levels of psychopathic personality traits, as highlighted above, we also assessed and compared self-reported psychopathic personality traits in the current samples of sexual and violent offenders, and non-offenders. We hypothesized that both sexual and violent offenders would show impairments in emotion recognition relative to non-offenders, and consistent with earlier findings, we hypothesized that these impairments would be particularly marked for expressions of negative affect (Hoaken et al., 2007; Robinson et al., 2012; Bagcioglu et al., 2014).

## Materials and Methods

### Ethical Approval

Ethical approval for this study was granted by the Committee for Ethical Review of the host University and the National Offender Management Service for the United Kingdom of England and Wales. We followed the principles for ethical conduct in research, outlined by the British Psychological Society, in all aspects of this work.

### Participants

Participants were 13 sexual offenders, 16 violent non-sex offenders, and 19 non-offending controls. All offending participants were recruited from the Therapeutic Community for adult male prisoners at HM Prison Grendon. Offense types for the sex offender group included rape or attempted rape of a child or an adult victim. The average age of the sex offender sample was 50.5 years (SD = 5.9), with a range of 40–62 years. Although the sample had a mixed history of treatment, the majority had completed the Sex Offender Treatment Program (*n* = 11) and/or Enhanced Thinking Skills (*n* = 10). Out of the 13 sex offenders tested, seven reported a history of early physical and/or emotional abuse. Offense types for violent offenders included murder and wounding with intent to do grievous bodily harm. Violent offenders had an average age of 37.8 years (SD = 10.4), with a range of 24–58 years. Violent offenders treatment histories included Enhanced Thinking Skills (*n* = 9), Counseling, Assessment, Referral Advice, Throughcare (*n* = 4), and the Prisoners – Addressing Substance Related Offending (P-ASRO) program (*n* = 3). A total of nine violent offenders reported a history of early physical and/or emotional abuse. We also tested a non-offending, community control group of 19 males with a mean age of 48.2 years (SD = 14.7), and a range of 26–67 years. Control participants were awarded £10 compensation for their time.

### Materials

#### Facial Expression Stimuli

**FIGURE 1:**
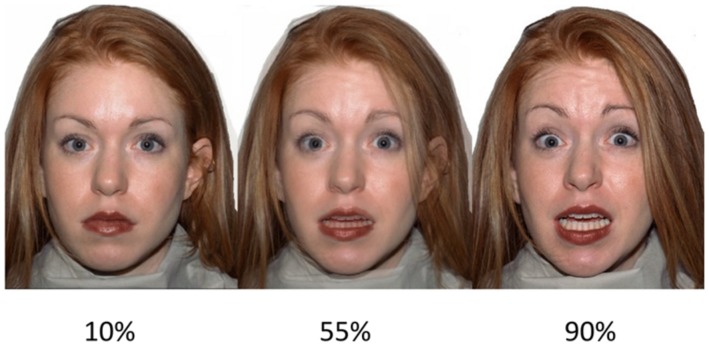
**Example stimuli. A female fearful expression expressed at (left–right)**: low intensity, moderate intensity, and high intensity.

Ten different Caucasian models (five females, five males) were selected from the NimStim Face Stimulus Set^1^. Models in the NimStim set are photographed showing each of seven different expressions: neutral, angry, disgust, fear, happy, sad, and surprise. The models were selected based on the NimStim validity data for expression recognition (Tottenham et al., 2009), which indicated good validity for the selected expressions (mean proportion correct, SD in brackets): angry = 0.85 (0.13), disgust = 0.85 (0.13), fear = 0.84 (0.13), happy = 0.85 (0.13), sad = 0.85 (0.13), surprised = 0.85 (0.13), neutral = 0.84 (0.13). In order to manipulate the intensity of the emotional expressions, each expression was morphed from neutral to 100% expressive in ten successive frames using the STOIK Morph Man software^2^. This resulted in ten morphed continua for each of the six emotional expressions for the 10 selected models. For task purposes, we selected three frames of varying intensity for each expression: low intensity (10% expressive), moderate intensity (55% expressive), and high intensity (90% expressive). Thus, we had 18 faces across all expressions for each model, 180 faces in total. Example of facial expression stimuli are shown in **Figure 1**.

#### Levenson Self Report Psychopathy Scale

The Levenson Self Report Psychopathy Scale (LSRP; Levenson et al., 1995) was developed for the assessment of psychopathic personality traits in non-institutionalized populations. The scale parallels the two factor structure of the Hare PCL-R (Hare, 1991, 2003), with moderate correlations observed for the primary and secondary subscales with Factors 1 and 2 of the PCL-R, respectively (Brinkley et al., 2001). The LSRP contains 26 items measured on a four-point Likert scale. Of these items, 16 items measure the ‘primary’ or affective/interpersonal psychopathic traits (e.g., lack of empathy, cunning, and manipulative), while the remaining items assess the ‘secondary’ or lifestyle/antisocial psychopathic traits (e.g., proneness to boredom, and impulsivity). Participants rate the degree to which they agree with each item on a scale of 1 = *Disagree Strongly*, to 4 = *Agree Strongly.* Levenson et al. (1995) have demonstrated adequate internal consistency for the LSRP, with a Cronbach’s alpha of 0.82 for the primary subscale, and 0.63 for the secondary subscale (considered adequate for a 10-item scale) in a sample of 487 undergraduate psychology students. All participants completed the LSRP.

#### Marlowe–Crowne C

Socially desirable responding was assessed using the Marlowe–Crowne Form C (MC-C; Reynolds, 1982). This short form scale includes 13 *true* or *false* items for the assessment of social desirability bias. Reynolds (1982) demonstrated acceptable internal consistency for the MC-C. A total of five participants (one sex offender and four violent offenders) failed to complete the Marlowe–Crowne Short Form C.

### Procedure

Stimuli were presented using E-Prime 2.0 stimulus presentation software on a Samsung Electronics laptop computer. Faces were presented in a random order and remained on screen while the participant made a response. Participants were asked to categorize each face as either neutral, or one of six universally recognized emotional expressions: anger, disgust, fear, happy, sad, or surprise, using the numeric keys zero to six, respectively. The expression labels were listed on the left hand side of the screen alongside the corresponding number key for each expression. There were 180 trails, each presenting a different stimulus varying in the model, expression, and intensity of the expression.

### Method for Analysis

Emotion recognition data were analyzed according to the principles of Signal Detection Theory (SDT; Green and Swets, 1966). Sensitivity was calculated as the discriminability index (*d*′), where increasing values of *d*′ refer to a greater sensitivity to a given signal or expression. Response bias was calculated as the criterion (*c*), with higher values of *c* indicative of a more conservative response style for a given emotion. In the current experiment, the data for the 55 and 90% intensities were used to compute the hit rate (HR) for a given emotion. Thus, HR was calculated as the proportion of moderate (55%) or high (90%) intensity expressions that were correctly classified as showing a given emotion, for male and female faces. The data for all of the 10% expressions were used to compute the false alarm (FA) rate. For these 10% faces it was assumed that the correct response is neutral. Thus, the FA rate was calculated as the proportion of low intensity (10%) male faces and female faces that were classified as showing a given emotion. HRs and FAs were adjusted to avoid extreme values of 0 and 1 using the loglinear approach of Hautus (1995), whereby 0.5 is added to the number of hits and the number of FAs, and 1 is added to the number of signal trials and the number of noise trials.

We calculated *d*′ based on commands provided by Stanislaw and Todorov (1999). The *d*′ measure was computed as the difference between the normalized *z* value of the HR and FA rate: (*d*′ = *z*HR – *z*FA). It represents a participant’s sensitivity to a given emotional expression independent of the participant’s bias toward labeling a neutral expression as that emotion. The *c* measure is computed as averaging the normalized *z* value of the HR and the normalized *z* value of the FA rate, then multiplying the result by negative one [*c* = –(*z*HR + *z*FA)/2].

Responses were analyzed using a mixed model ANOVA on *d*′ and *c*, with the between subjects factor group (non-offenders, sexual offenders, violent offenders), and the following with-in subject factors: sex of model (male, female); expression intensity (moderate, high); and emotion expressed (anger, disgust, fear, happy, sad, surprise). Significant interactions resulting from the analysis were broken down using further ANOVAs to examine, for example, effects for female faces and male faces separately. This strategy meant that all further analyses were guided by the results of the initial mixed ANOVA. All significant *F* tests on group were further examined using Fisher’s LSD *post hoc* tests, as recommended by Keselman (1998) for *k* = 3 independent groups. Statistical analyses were carried out using IBM SPSS Statistics 20 for Microsoft Windows. Effect sizes for ANOVA are reported as partial-eta squared with the following suggested norms for interpretation: small = 0.01; medium = 0.06; large = 0.14 (Cohen, 1988).

## Results

### Psychopathic Trait Scores

**Table 1** shows mean scores for non-offenders, sexual offenders, and violent offenders on all questionnaires. We observed a significant, positive correlation of primary, and secondary psychopathic traits across all participants (*r* = 0.45, *p* = 0.001). A series of independent ANOVAs showed that the three groups did not differ in primary psychopathic traits *F*(2,45) = 0.78, *p* > 0.05, $\eta_{p}^{2}$= 0.03, total psychopathic traits *F*(2,45) = 1.77, *p* > 0.05, $\eta_{p}^{2}$= 0.07, or socially desirable responding *F*(2,45) = 2.23, *p* > 0.05, $\eta_{p}^{2}$= 0.10. However, the groups differed significantly in levels of secondary psychopathic traits *F*(2,45) = 3.61, *p* < 0.05, $\eta_{p}^{2}$= 0.14, with higher scores among violent offenders compared with sexual-offenders and non-offenders (*p* < 0.05). The difference in secondary psychopathic traits between sexual offenders and non-offenders was non-significant.

**Table 1:** Levels of psychopathic traits and socially desirable responding in non-offenders, sexual offenders, and violent offenders.

	Non-offenders (*n* = *19)*	Sexual offenders (*n* = *13)*	Violent offenders (*n* = *16)*
	
Measure	Mean (SD)		
LSRP-P	29.3 (7.2)	26.5 (7.2)	29.9 (8.6)
LSRP-S	19.4 (3.2)^a^	19.2 (5.9)^a^	23.1 (4.9)^b^
LSRP-Total	48.6 (2.1)	45.8 (11.0)	53.1 (11.8)
MC-C	7.3 (2.3)	5.8 (3.4)	5.4 (2.4)

*LSRP-P, primary subscale of the Levenson Self Report Psychopathy Scale; LSRP-S, secondary subscale of the Levenson Self Report Psychopathy Scale; MC-C, Marlowe–Crowne Short Form C. Scores that are significantly different share different superscript letters (*p* < 0.05).*

Primary and secondary psychopathy scores for non-offenders in the present study were within the range reported by others for non-offending samples, with primary psychopathy scores in earlier studies found to range from 28 to 35, and secondary psychopathy from 20 to 23 (Levenson et al., 1995; Campbell et al., 2009; Gummelt et al., 2012). We also compared levels of psychopathic traits in this study with those obtained by Brinkley et al. (2001) for the primary (*M* = 32.99, SD = 8.19) and secondary (*M* = 21.68, SD = 5.05) subscales of the LSRP, in a sample of 549 male inmates from Wisconsin state prisons. We used mean values and pooled SD to calculate Cohen’s *d* effect sizes, with the following suggested norms for interpretation: small = 0.2; medium = 0.5; and large = 0.8 (Cohen, 1988). Comparisons revealed lower levels of primary psychopathic traits among our non-offender (*d* = 0.40), and sexual (*d* = 0.80) and violent (*d* = 0.38) offender samples relative to the sample of Brinkley et al. (2001). We also found lower levels of secondary psychopathic traits among our non-offender (*d* = 0.46) and sexual offender (*d* = 0.49) samples. However, scores for secondary psychopathic trait scores for the current sample of violent offenders were slightly elevated relative to Brinkley et al. (2001; *d* = 0.28). Thus, we observed small to large sized effects for primary psychopathic traits, with lower scores observed in this study compared with that of Brinkley et al. (2001). However, secondary psychopathic trait scores were slightly elevated in the current sample of violent offenders compared to the sample of Brinkley et al. (2001).

### Sensitivity (d′)

**FIGURE 2:**
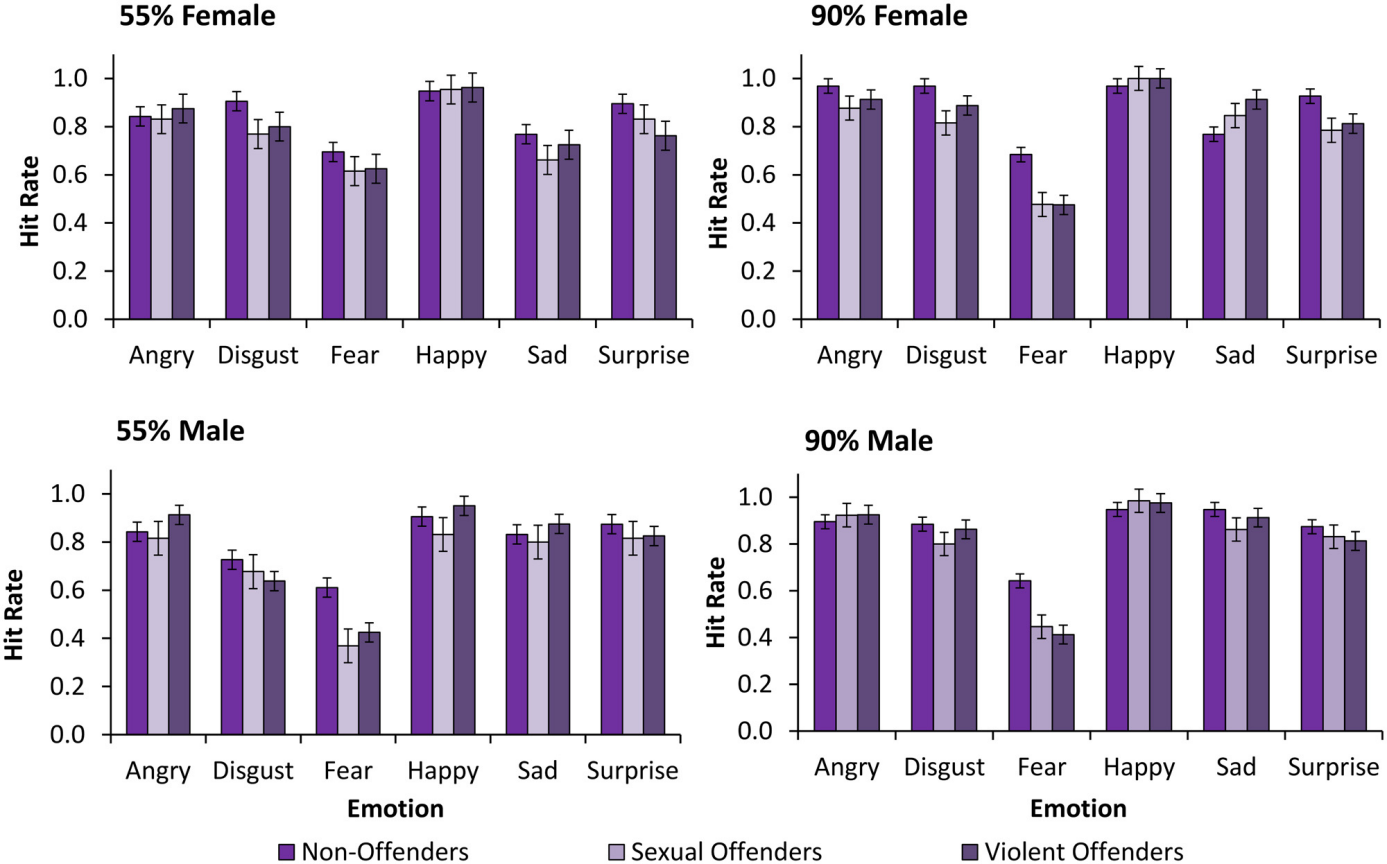
**Hit rates (HR)**. HR are shown by emotion expressed for female faces at moderate intensity, female faces at high intensity, male faces at moderate intensity, and male faces at high intensity, for non-offenders, sexual offenders, and violent offenders. Error bars = SE mean.

**FIGURE 3:**
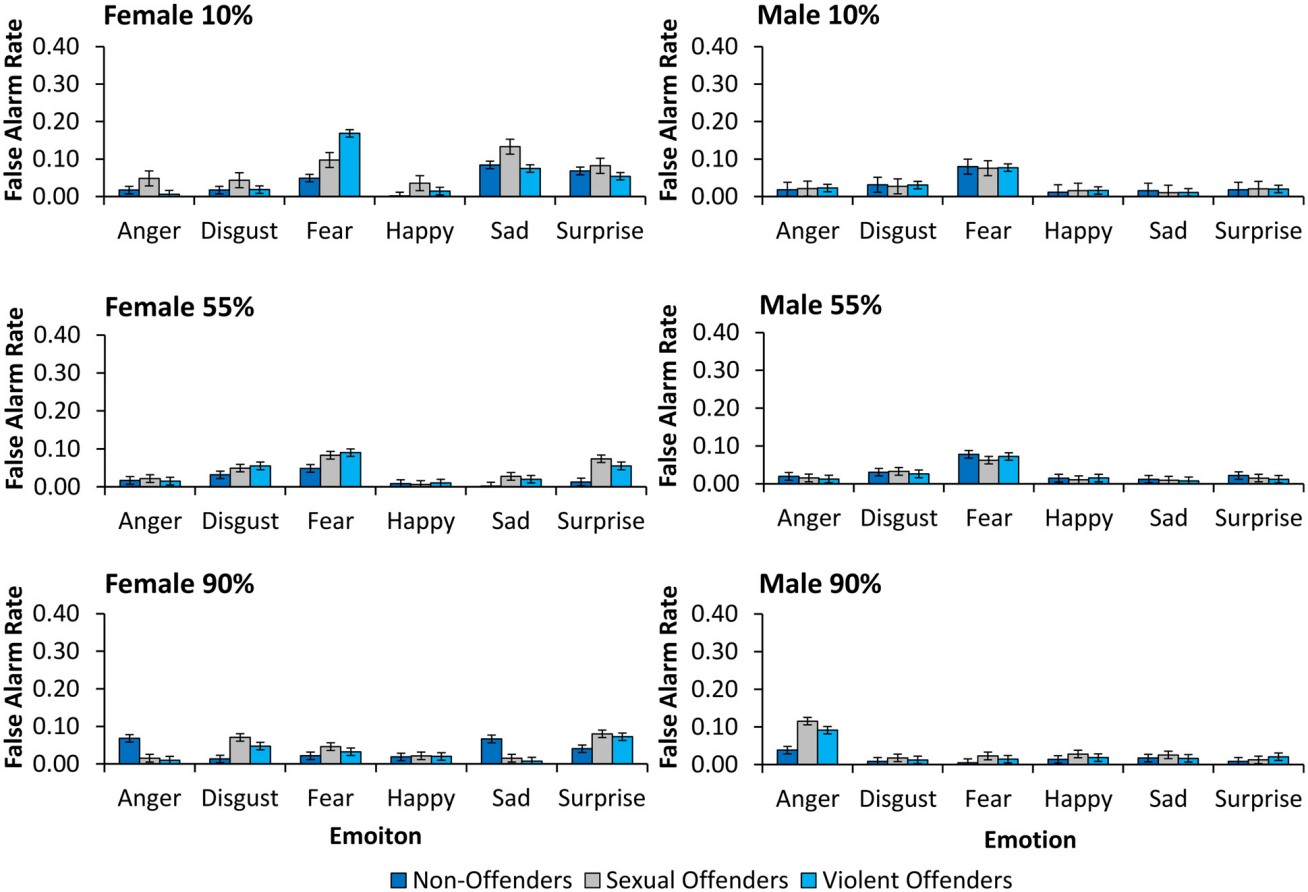
**False alarms (FAs)**. FAs are shown by classification type for female faces at low intensity, male faces at low intensity, female faces at moderate intensity, male faces at moderate intensity, female faces at high intensity, and male faces at high intensity, for non-offenders, sexual offenders, and violent offenders. Error bars = SE mean.

For descriptive purposes, in **Figure 2** we show HR data for non-offenders, and sexual and violent offenders, for each emotion, displayed by female and male faces, at moderate (55%) and high (90%) intensity. **Figure 3** shows FA data for non-offenders, and sexual and violent offenders, as a function of the misclassification type (emotion attributed) for female and male faces at low (10%), moderate (55%), and high (90%) intensity. However, it should be noted that *d*′ and *c* were calculated on the basis of HR data for male and female expressions at moderate and high intensity, and FA data for only low intensity female and male faces.

**Table 2** shows sensitivity (*d*′) for each expression, for male and female faces, at moderate (55%) and high (90%) intensity, for non-offenders, and sexual and violent offenders. The analysis on *d*′ revealed a trend toward differences in overall sensitivity between the three groups *F*(5,225) = 2.94, *p* = 0.063, $\eta_{p}^{2}$= 0.12, with non-offenders showing greater sensitivity compared with sexual offenders (*p* = 0.02). There were no significant differences in sensitivity for non-offenders and violent offenders (*p* = 0.35), or for violent offenders and sexual offenders (*p* = 0.15).

**Table 2:** Discriminability index (*d*′) for non-offenders, and sexual and violent offenders, for each expression displayed at moderate (55%) and high (90%) intensity, by female and male faces.

		*d*′		
		Non-offenders *M* (SE)	Sexual offenders *M* (SE)	Violent offenders *M* (SE)
Female 55%	Angry	2.86 (0.13)	2.56 (0.23)	3.09 (0.14)
	Disgust	3.09 (0.11)	2.48 (0.26)	2.78 (0.22)
	Fear	2.26 (0.17)	1.65 (0.36)	1.66 (0.27)
	Happy	3.34 (0.10)	3.09 (0.17)	3.25 (0.12)
	Sad	2.25 (0.18)	1.57 (0.27)	2.08 (0.19)
	Surprise	2.65 (0.17)	2.36 (0.28)	2.49 (0.29)
Male 55%	Angry	2.72 (0.17)	2.68 (0.26)	2.89 (0.22)
	Disgust	2.50 (0.10)	2.16 (0.23)	2.35 (0.10)
	Fear	2.32 (0.17)	1.34 (0.26)	1.75 (0.20)
	Happy	3.17 (0.10)	2.72 (0.20)	3.18 (0.11)
	Sad	2.39 (0.19)	2.20 (0.28)	2.46 (0.15)
	Surprise	2.81 (0.14)	2.58 (0.27)	2.77 (0.23)
Female 90%	Angry	3.24 (0.09)	2.68 (0.24)	3.22 (0.14)
	Disgust	3.30 (0.10)	2.63 (0.22)	3.03 (0.17)
	Fear	2.23 (0.20)	1.32 (0.30)	1.26 (0.34)
	Happy	3.42 (0.09)	3.25 (0.14)	3.37 (0.08)
	Sad	2.24 (0.24)	2.07 (0.19)	2.60 (0.16)
	Surprise	2.77 (0.17)	2.28 (0.31)	2.59 (0.23)
Male 90%	Angry	2.89 (0.14)	2.99 (0.22)	2.91 (0.18)
	Disgust	2.97 (0.12)	2.48 (0.30)	2.99 (0.12)
	Fear	2.38 (0.15)	1.55 (0.27)	1.70 (0.19)
	Happy	3.29 (0.07)	3.16 (0.14)	3.27 (0.09)
	Sad	2.74 (0.18)	2.38 (0.25)	2.61 (0.19)
	Surprise	2.82 (0.12)	2.64 (0.25)	2.71 (0.22)

We also observed a marginally significant interaction of expression and group *F*(10,225) = 1.83, *p* = .057, $\eta_{p}^{2}$= 0.08. When broken down by emotion, we found a significant effect of group for fearful expressions *F*(2,45) = 5.05, *p* = 0.011, $\eta_{p}^{2}$= 0.18, with non-offenders showing greater sensitivity to fearful expressions compared with sexual (*p* = 0.007) and violent (*p* = 0.015) offenders. There were no differences in sensitivity to fearful expressions between sexual and violent offenders (*p* = 0.679). The effect of group was also significant for sensitivity to disgust expressions *F*(2,45) = 3.74, *p* = 0.032, $\eta_{p}^{2}$= 0.14, with non-offenders showing greater sensitivity compared with sexual offenders (*p* = 0.009). There was also a trend for greater sensitivity to disgust expressions among violent compared with sexual offenders (*p* = 0.089), while the difference between controls and violent offenders was non-significant (*p* = 0.333). The effect of group was non-significant for angry, happy, sad, and surprised expressions (all *F* < 1.40, *p* > 0.20).

We also found a significant interaction of group with intensity, sex, and expression *F*(10,225) = 1.96, *p* = 0.039, $\eta_{p}^{2}$= 0.08. To better understand this interaction, we analyzed sensitivity for female faces and male faces separately. The interaction of group with intensity and expression for male faces was non-significant (*p* = 0.438). However, we showed that group interacted with intensity and expression for female faces *F*(10,225) = 2.03, *p* = 0.031, $\eta_{p}^{2}$= 0.08. Although the interaction of group and expression was non-significant for female faces at moderate intensity (*p* = 0.471), there was a significant interaction of group and expression for female faces at high intensity *F*(10,225) = 2.03, *p* = 0.031, $\eta_{p}^{2}$= 0.08.

An analysis for each female expression at high intensity revealed a significant effect of group for sensitivity to angry expressions *F*(2,45) = 3.92, *p* = 0.027, $\eta_{p}^{2}$= 0.15, with sexual offenders showing reduced sensitivity compared with non-offenders (*p* = 0.014), and violent offenders (*p* = 0.021). The difference between non-offenders and violent offenders was non-significant (*p* = 0.919). We also found a significant effect of group for sensitivity to disgust expressions *F*(2,45) = 4.37, *p* = 0.018, $\eta_{p}^{2}$= 0.16, with greater sensitivity among non-offenders compared with sexual offenders (*p* = 0.005). However, the difference in sensitivity for violent offenders compared with sexual offenders (*p* = 0.095), and non-offenders (*p* = 0.215), was non-significant. Finally, we found that there was a significant effect of group on sensitivity to fearful expressions *F*(2,45) = 4.09, *p* = 0.023, $\eta_{p}^{2}$= 0.15, with lower sensitivity among both sexual (*p* = 0.029) and violent (*p* = 0.014) offenders compared with non-offenders. However, both sexual offenders and violent offenders showed similar levels of sensitivity (*p* = 0.888). There were no significant group differences in sensitivity to happy, sad, or surprised expressions (all *p* > 0.21).

To summarize, we found that sexual and violent offenders showed marginally reduced sensitivity to emotional expressions across intensity and sex, compared with non-offenders. We also found particularly impaired sensitivity for fearful expressions among sexual and violent offenders compared with non-offenders, while sexual offenders were generally impaired for disgust compared with non-offenders. We also found particularly pronounced differences between groups for high intensity female expressions, with sexual offenders showing reduced sensitivity to anger compared with violent and non-offenders, and reduced sensitivity to disgust compared with non-offenders. Furthermore, both violent and sexual offenders were impaired relative to non-offenders for high intensity female fear expressions.

### Criterion (*c*)

**Table 3** shows criterion *(c)* for each expression, for male and female faces, at moderate (55%) and high (90%) intensity, for non-offenders, and sexual and violent offenders. An ANOVA on *c* showed a significant interaction of group with intensity, sex, and expression *F*(5,225) = 1.96, *p* = 0.039, $\eta_{p}^{2}$= 0.08. This interaction was most readily broken down by group, with a significant interaction of intensity, sex, and expression among violent offenders only *F*(5,75) = 2.72, *p* = 0.026, $\eta_{p}^{2}$= 0.15. For violent offenders, we found a significant interaction of sex and expression for moderate intensity (55%) *F*(5,75) = 8.41, *p* < 0.001, $\eta_{p}^{2}$= 0.36, and high intensity (90%) faces *F*(5,75) = 2.72, *p* = 0.026, $\eta_{p}^{2}$= 0.15. For moderate intensity faces, violent offenders showed a significant effect of expression for male faces *F*(5,75) = 14.05, *p* < 0.001, $\eta_{p}^{2}$= 0.48, but not female faces (*p* = 0.672). Bonferroni adjusted *post hoc* tests showed that for moderate intensity male faces, violent offenders showed a more conservative response style for classifying faces as disgust or fear, compared with angry, happy, or sad (all *p* < 0.01). For high intensity faces, violent offenders again showed a significant effect of expression for male faces *F*(5,75) = 17.00, *p* < 0.001, $\eta_{p}^{2}$= 0.53. Bonferroni adjusted comparisons again revealed a more conservative response style for fear compared with all other emotions (*p* < 0.01), as well as a lower criterion for labeling faces as sad compared with disgust and surprise (*p* < 0.05). Although there was also a significant effect of expression for high intensity female faces *F*(5,75) = 2.99, *p* = 0.016, $\eta_{p}^{2}$= 0.17, Bonferroni adjusted *post hoc* tests failed to reach significance for any of the comparisons.

**Table 3:** Criterion (*c*) for non-offenders, and sexual and violent offenders, for each expression displayed at moderate (55%) and high (90%) intensity, by female and male faces.

		*c*		
		Non-offenders	Sexual offenders	Violent offenders
		*M* (SE)	*M* (SE)	*M* (SE)
Female 55%	Angry	0.54 (0.08)	0.42 (0.09)	0.54 (0.08)
	Disgust	0.48 (0.07)	0.54 (0.10)	0.59 (0.07)
	Fear	0.63 (0.11)	0.55 (0.11)	0.49 (0.17)
	Happy	0.45 (0.05)	0.33 (0.08)	0.36 (0.17)
	Sad	0.44 (0.11)	0.43 (0.11)	0.47 (0.10)
	Surprise	0.28 (0.08)	0.32 (0.08)	0.54 (0.09)
Male 55%	Angry	0.52 (0.07)	0.49 (0.11)	0.34 (0.09)
	Disgust	0.72 (0.08)	0.64 (0.11)	0.87 (0.06)
	Fear	0.86 (0.10)	1.00 (0.10)	1.06 (0.11)
	Happy	0.51 (0.06)	0.47 (0.33)	0.39 (0.06)
	Sad	0.35 (0.09)	0.33 (0.08)	0.29 (0.07)
	Surprise	0.46 (0.06)	0.48 (0.09)	0.52 (0.09)
Female 90%	Angry	0.35 (0.06)	0.35 (0.06)	0.47 (0.08)
	Disgust	0.38 (0.06)	0.46 (0.12)	0.47 (0.08)
	Fear	0.64 (0.09)	0.71 (0.09)	0.68 (0.17)
	Happy	0.41 (0.04)	0.24 (0.07)	0.30 (0.04)
	Sad	0.45 (0.06)	0.18 (0.10)	0.21 (0.08)
	Surprise	0.22 (0.08)	0.36 (0.10)	0.49 (0.08)
Male 90%	Angry	0.44 (0.09)	0.34 (0.09)	0.34 (0.10)
	Disgust	0.49 (0.08)	0.48 (0.09)	0.55 (0.08)
	Fear	0.83 (0.11)	0.89 (0.10)	1.08 (0.09)
	Happy	0.45 (0.04)	0.25 (.08)	0.34 (0.05)
	Sad	0.18 (0.08)	0.24 (0.08)	0.22 (0.06)
	Surprise	0.45 (0.07)	0.46 (0.10)	0.56 (0.08)

To summarize, violent offenders showed a higher criterion for classifying moderate intensity male faces as disgust or fear, compared with angry, happy, or sad. Violent offenders also showed a higher criterion for labeling high intensity male faces as afraid compared with all other emotions, but were more liberal when labeling faces as sad compared with disgust and surprise. These results suggest that violent offenders show particular biases when labeling the emotions of other males, and are particularly conservative for labeling both moderate and high intensity expressions as fearful.

## Discussion

The aim of this study was to examine sensitivity to emotional facial expressions among both sexual and violent offenders, and non-offenders. It was hypothesized that both types of offender would show reduced sensitivity to emotional expressions compared with non-offenders, and that these difficulties would be particularly marked for negative expressions. Compared with non-offenders, sexual offenders showed marginally reduced sensitivity to emotional expressions across expression type, intensity, and sex. We also found evidence for marginally reduced sensitivity to fearful expressions among both sexual and violent offenders compared with non-offenders, and reduced sensitivity to expressions of disgust among sexual offenders compared with non-offenders. These findings are consistent with the results of Hoaken et al. (2007) who found evidence for impaired emotion recognition in a sample of violent offenders that included sexual offenders, relative to non-violent offenders and non-offenders. Similarly, Gery et al. (2009) observed emotion recognition impairments among sexual offenders compared with non-sexual, non-violent offenders, and non-offending controls. Taken together, these findings suggest that emotion recognition impairments appear to be a common characteristic observed among both violent and sexually violent offenders.

More specific findings from this study showed that both sexual and violent offenders, compared with non-offenders, were less sensitive to female fearful expressions at high intensities. These findings are consistent with the results of a meta-analysis from Marsh and Blair (2008) that showed evidence for a marked impairment in fearful face recognition among instrumentally violent populations. It is argued by Blair (1995, 2001, 2003) that a failure to recognize others distress signals as aversive, including the expressions of fear and sadness, may lead to a breakdown in the development of affective empathy. Furthermore, Blair (1995, 2001, 2003) argues that this breakdown may be associated with problems inhibiting aggressive behaviors in response to other’s submissive cues. Thus, the finding of impaired fearful expression recognition among sexual offenders is consistent with conceptual models that cite deficiencies in violence inhibition in the etiology of sexual offending (Marshall and Barbaree, 1990; Barbaree and Marshall, 1991).

Other sensitivity impairments for high intensity female faces showed that sexual offenders were less sensitive to angry expressions, compared with violent offenders and non-offenders, and less sensitive to disgust relative to non-offenders. These findings support the hypothesis that sensitivity impairments would be particularly pronounced for negative expressions, and are consistent with the finding of reduced accuracy for angry, disgust, and fearful expressions among sexual offenders compared with non-sexual, non-violent offenders (Gery et al., 2009). Furthermore, the finding of impaired female disgust recognition suggests that sexual offenders may show problems in interpreting females’ aversion to a range of disgust inducing stimuli (Rozin et al., 1993), including bodily products, sex, and body envelope violations (Haidt et al., 1994).

The implication that sexual offenders may misinterpret females’ social cues is consistent with results showing that sexually aggressive men show difficulty recognizing indicators of negative mood (Lipton et al., 1987), and tend to misinterpret female’s friendliness as seductiveness (Malamuth and Brown, 1994). The finding of differences in the processing of female and male expressions is consistent with the observation of differential processing of male and female threat related bodily postures among non-offenders (Kret and de Gelder, 2013), and suggests that differences in the expressivity of male and female faces (Schwartz et al., 1980; Brody and Hall, 1993), and the relative ease with which male and female expressions can be recognized (Hess et al., 1997), represent important variables for consideration in research with offending populations.

Despite finding problems in emotion recognition among sexual and violent offenders, it remains unclear how these difficulties relate to specific types of sexual offending behavior. For example, offenders convicted of grooming young children may show an intact ability to recognize others emotions, and this ability may aid the grooming process. Alternatively, some rapists have been reported to enjoy watching their victims in pain (Kirsch and Becker, 2007). These individuals may therefore show a preserved ability to process others distress signals. Potential differences in social cognition between different types of sexual offender may relate to differences in the motivations and characteristics of sexual offenders with adult and child victims. For example, Mitchell and Beech (2011) review literature showing that while pedophilic sexual offenders show intimacy deficits and elevated levels of social phobia, rapists of adult women are more likely to show a generally antisocial behavioral pattern and have high levels of psychopathic traits. These characteristics may be related to different types of social cognitive impairment. However, while it may be of empirical interest to examine differences in emotional expression recognition between sexual offenders with child and adult victims, the limited sample size for sexual offenders in the present study precludes performing separate analyses based on victim age.

As well as sensitivity, we also examined the extent to which sexual and violent offenders differed from non-offenders in the extent to which they showed a more liberal, or a more conservative response style when labeling emotional faces. Previous studies have found evidence for a hostile attribution bias among children with conduct problems and antisocial behavior (Dodge and Coie, 1987; Crick and Dodge, 1996). In this study we observed particular response styles among violent offenders that were dependent upon the sex and the intensity of the expression. In particular, for moderate intensity male faces, violent offenders showed a higher criterion for classifying expressions as disgust or fear, compared with angry, happy, or sad. Similarly, for high intensity male faces, violent offenders showed a more conservative response style for labeling expressions as fear compared with all other emotions, and were more liberal for labeling faces as sad compared with disgust or surprise. Although these findings do not show direct support for a hostile attribution bias among violent offenders, they are nonetheless consistent with the observation of particular biases in facial emotion recognition among antisocial populations. These findings suggest that violent offenders are more conservative when labeling male faces as afraid compared with other emotions, and this bias may contribute to failures to recognize and appropriately respond to others submissive cues.

The findings from this study may have been influenced by considerable variability in the age of participants, ranging from 24 to 67 across the three groups, with impaired processing of emotional expressions previously noted among elderly participants relative to the young (Sullivan et al., 2007). Results may also have been influenced by the potential presence of psychological disorders, including autism, and schizophrenia, that are known to effect the processing of emotional expressions but were not formally assessed in the current study. In relation to psychopathy, we showed that there were no differences between offenders and non-offenders in levels of primary psychopathic traits. However, secondary psychopathic traits were found to be elevated among violent compared with sexual and non-offenders. However, it is theorized that psychopathy related difficulties in emotional expression recognition are more strongly related to the interpersonal and affective features of the disorder, rather than the lifestyle and antisocial features (Blair, 1995, 2001). Despite potential pitfalls in the self-report measurement of psychopathy, including the risk of biased and dishonest reporting (Lilienfeld and Fowler, 2006), findings from a meta-analysis show that self-report instruments nonetheless provide a reliable indicator of psychopathic traits (Ray et al., 2013). Finally, although limited sample sizes in this study may have implications for statistical power and the interpretation of effects, moderate to large effect sizes reported here are nonetheless indicative of meaningful differences in expression recognition between offenders and non-offenders.

These findings may be indicative of a need to target the processing of facial expression information in treatment programs for sexual and violent offenders. Indeed, the instruction to attend toward the eye region of emotional faces can lead to improvements in emotion recognition among children with callous-unemotional traits (Dadds et al., 2008). Moreover, improvements in empathy and conduct problems have also been observed among high callous-unemotional traits children following a randomized controlled trial of emotion recognition training (Dadds et al., 2012). However, although training may lead to improvements in emotion recognition, it has been cautioned that such improvements may not necessarily equate to improvements in affective empathy, and that outcome measures following emotion recognition training should be coupled with the measurement of relevant psychophysiological parameters (Gillespie et al., 2014). The results of this study support the consistent finding of emotion recognition impairments among antisocial populations, and extend these findings to show that both sexual and violent offenders present with particular patterns of emotion recognition impairments compared with non-offenders. Difficulties in expression recognition may therefore represent an etiological factor in the offending behavior of both types of offender. Furthermore, violent offenders also showed a conservative response style for labeling male faces as fearful compared with other emotions. Although these findings should be extended to include a larger sample with distinct groups of sexual offenders with child and adult victims, they are nonetheless indicative of abnormalities in the processing of social cognitive information among both sexual and violent offenders.

## Conflict of Interest Statement

The authors declare that the research was conducted in the absence of any commercial or financial relationships that could be construed as a potential conflict of interest.

## Footnotes

1 http://www.macbrain.org/resources.htm

2 http://www.stoik.com/products/video/STOIK-MorphMan/
